# Draft Genome Sequence of Lactobacillus rhamnosus OSU-PECh-69, a Cheese Isolate with Antibacterial Activity

**DOI:** 10.1128/MRA.00803-20

**Published:** 2020-09-10

**Authors:** Israel García-Cano, Walaa E. Hussein, Diana Rocha-Mendoza, Ahmed E. Yousef, Rafael Jiménez-Flores

**Affiliations:** a Department of Food Science and Technology, The Ohio State University, Columbus, Ohio, USA; Georgia Institute of Technology

## Abstract

The novel strain Lactobacillus rhamnosus OSU-PECh-69 was isolated from provolone cheese. It produces antimicrobial agents having a molecular mass of 5 to 10 kDa that are active against Gram-positive and Gram-negative bacteria. The strain has a genome sequence of 3,057,669 bp, a GC content of 46.6%, and up to two gene clusters encoding bacteriocins.

## ANNOUNCEMENT

A new strain, designated OSU-PECh-69, was isolated from provolone cheese and identified by 16S rRNA gene sequencing as Lactobacillus rhamnosus (1). This species is a nonstarter lactic acid bacterium known to play an essential role in developing the final characteristics of cheeses during ripening (2). Grown on cheese acid whey-based growth medium, OSU-PECh-69 exhibited antimicrobial activity against several foodborne pathogens. After fermentation, antimicrobial agents with a molecular mass of 5 to 10 kDa were found in the ultrafiltrate fraction (3). The components of the fraction were analyzed further by liquid chromatography, coupled with mass spectrometry (3).

The availability of the draft genome sequence of the strain facilitates the identification of the putative genes encoding prospective antimicrobial compounds (4). The total genomic DNA (gDNA) was extracted from a single isolated colony grown in MRS broth (BD Difco, USA) using a Wizard genomic DNA purification kit (Promega, USA). The gDNA concentration was determined by the PicoGreen method (catalog number P7589; Life Technologies, USA) using a Victor X2 fluorometer (PerkinElmer, USA). A sequence library was constructed by random fragmentation of the gDNA followed by 5′ and 3′ adapter ligation (TruSeq DNA PCR-free library prep kit with 350-bp inserts; Illumina, USA). To verify the size of the adapter-ligated fragments, PCR analysis was performed using a 2200 TapeStation system (Agilent, USA). A NovaSeq 6000 S4 DNA sequencer (Illumina) was used to generate clonal clusters from the library through bridge amplification and to apply sequencing-by-synthesis technology (The Ohio State University Nucleic Acid Shared Resource Facility, Columbus, OH, USA).

The total number of read bases were analyzed using real-time analysis software (Illumina). The binary base call (BCL) format was converted to FASTQ files using bcl2fastq conversion software (Illumina). The raw read quality (score of 36) was determined using FastQC 0.11.8 (https://www.bioinformatics.babraham.ac.uk/projects/fastqc/), followed by quality trimming and filtering using BBDuk (https://jgi.doe.gov/data-and-tools/bbtools/bb-tools-user-guide/bbduk-guide/) and *de novo* assembly using MEGAHIT 1.2.8 (5). The quality of the assembled genome was assessed using QUAST 5.0.2 (6). The assembly generated 558 contigs (maximum size, 264,166 bp; minimum size, 205 bp) with a coverage of 27.3×, revealing a genome size of 3,057,669 bp, an *N*_50_ value of 91,528 bp, and an average GC content of 46.6%. The taxonomic classification of the assembled genome was determined using GTDB-Tk 1.0.2 (7), and its average nucleotide identity (ANI) with closely related genomes was calculated using FastANI 2.0 (8). Based on this analysis, the genome of OSU-PECh-69 shared the highest (97.57%) ANI with that of L. rhamnosus DSM 20021 (GenBank accession number GCA_000615245.1); thus, DSM 20021 was used as a reference genome in contig reordering, using progressiveMauve 2.4.0 (9). Default parameters were used for all software programs. Draft genome annotation was performed using the NCBI Prokaryotic Genome Annotation Pipeline (10), which identified 2,796 coding sequences, 4 noncoding RNAs (ncRNAs), 58 tRNAs, 10 rRNA genes, and 275 pseudogenes. Analysis of secondary metabolite biosynthesis gene clusters using antiSMASH 5.1 (11) and BAGEL 4 (12) revealed one and two clusters encoding potential bacteriocins, respectively.

### Data availability.

The draft genome sequence of Lactobacillus rhamnosus OSU-PECh-69 was deposited in the NCBI database under the BioProject accession number PRJNA634258. The Sequence Read Archive (SRA) number is SRR12450178. The GenBank whole-genome sequence accession number is JABURW000000000.
